# Regulating N Application for Rice Yield and Sustainable Eco-Agro Development in the Upper Reaches of Yellow River Basin, China

**DOI:** 10.1155/2014/239279

**Published:** 2014-06-17

**Authors:** Aiping Zhang, Ruliang Liu, Ji Gao, Shiqi Yang, Zhe Chen

**Affiliations:** ^1^Institute of Agro-Environment and Sustainable Development, CAAS/Key Laboratory of Agro-Environment and Climate Change, Ministry of Agriculture, Beijing 100081, China; ^2^Ningxia Academy of Agriculture and Forestry Sciences, Ningxia 75000, China

## Abstract

High N fertilizer and flooding irrigation applied to rice on anthropogenic-alluvial soil often result in N leaching and low recovery of applied fertilizer N from the rice fields in Ningxia irrigation region in the upper reaches of the Yellow River, which threatens ecological environment, food security, and sustainable agricultural development. This paper reported the regulating N application for rice yield and sustainable Eco-Agro development in the upper reaches of Yellow River basin. The results showed that reducing and postponing N application could maintain crop yields while substantially reducing N leaching losses to the environment and improving the nitrogen use efficiency. Considering the high food production, the minimum environmental threat, and the low labor input, we suggested that regulating N application is an important measure to help sustainable agricultural development in this region.

## 1. Introduction

The large world population creates a huge demand for food. To meet the challenge, more grains must be produced which requires more chemical fertilizer nitrogen. From 1960 to 2000, the use of nitrogen fertilizers increased by 800%, with wheat, rice, and maize accounting for almost half of current fertilizer use [1]. At 81.7 million tons (Mt), chemical fertilizer nitrogen (N) accounts for approximately half of all N reaching global croplands today and supplies basic food needs for at least 40% of the population [2]. As the largest consumer of chemical N in the world, China accounts for 32% of the world's total consumption, and approximately 18% of the chemical N is applied to rice paddies [3]. However, the nitrogen use efficiency is typically below 40% with these crops, indicating that most applied fertilizer is either washed out of root zone, which will result in increased N losses as leachate to freshwater and marine systems [4–7].

Located in the upper reaches of the Yellow River, Ningxia Irrigation Region with an irrigation area of 9697 km^2^ is one of the oldest and largest irrigation areas in northwest China and sustains over 60% of the Ningxia population. From 1980 to 2011, total annual grain production increased from 1.20 to 3.59 billion kg (an increase of 2.99 times), and chemical fertilizer application increased from 171,000 tons to 1033,000 tons (an increase of 6.04 times), of which chemical N fertilizer application was 533,000 tons [8] (Ningxia Statistical Yearbook, 2012). Ningxia Irrigation Region is located in the arid and semiarid zones, with the average annual precipitation of 180–220 mm, which is extremely lower compared to the 1000–1550 mm evaporation. The fertile alluvial soil is formed by accumulated sediments transferred by the Yellow River with abundant transboundary water about 32.5 billion m^3^ in Ningxia segment annually. In this region, agriculture essentially relies on irrigation water. About 7 billion m^3^ water from the Yellow River is drawn and 2.5 billion m^3^ is returned annually [9] (Ningxia Water Source Bulletin, 2008). Over 93–95% of the water is used for agriculture. Rice is a major crop grown in the anthropogenic-alluvial soil and is often perceived as the largest water and fertilizer consumer in the basin. In the rice fields, the application rate of fertilizer is as high as 300 kg ha^−1^ due to flooding irrigation. Large amounts of N fertilizer into the land will inevitably leach N into the water bodies, and thus becoming one of the nonpoint source pollution to the Yellow River.

Cui et al. [10] surveyed the water quality between the upstream station where the Yellow River flows in Ningxia boundary and the downstream station where Yellow River flows out Ningxia boundary during the 2005-2006. He estimated that N washed out from farmland into the Yellow River was 41.1 thousand tons, which is 1.52 times higher than those from the point source pollution during the same time period. de Data and Buresh [11] analyzed the correlation between N fertilizer application rates and total N contents of the river water and found that N contents in outboundary station in the downstream reaches coincidently increased with the N application rates in the Irrigation Region. Fixen and West [12] found that both total N and ammonium N contents were increasing significantly with the increase of fertilizer application rates, especially since 1990s in Ningxia segment of the Yellow River. Total N and ammonium N contents in water samples taken from the outboundary station were obviously higher than those from the upstream station. This clearly indicates the influence of fertilizer application in the Irrigation Region on the water quality of the Yellow River. Zhang et al. [13] also demonstrated that fertilizer and pesticide were the main nonpoint source pollutants of underground water.

Nitrogen leaching from agricultural soils can represent a substantial loss of fertilizer N and put pressure on the surrounding environment. Studies have already showed that N discharged into the Yellow River in Ningxia Irrigation Region was estimated to be 20–65% of the total N lost from the fertilizer [11], and the annual N loss from the rice field in Region was 28,865 tons [14]. The challenge is to continue to help meet food need while minimizing the risk of negative environmental impacts through improved N use efficiency [15–19].

In Ningxia, the pattern applying N fertilizer to a single-seasonal rice consists mostly of 50~70% base fertilization and 50~30% green-turning and tillering fertilization. Traditionally, farmers hold that topdressing additional N fertilizer in the booting stage would make for the fact that the paddy remains green when it is due to become yellow and ripe. The truth is that application of excessive base N fertilizer would not only make it hard to meet the demand of the currently prevailing high-yield breed of rice on nitrogen but also lead to more nitrogen loss from leaching because rice needs less N-nutrition for its growth before the jointing stage [20–23].

Therefore, there is a need for looking for optimizing N application amount and time that will improve N efficiency and reduce N leaching loss while maintaining rice yield. The objectives of this study were (1) to quantify the leaching amount of inorganic N during different rice growth stages as affected by RPN and (2) to estimate the effects of RPN on N use efficiency and rice yields (Table 3). We hope to recommend alternative N application techniques that will reduce N losses while maintaining crop yields.

## 2. Materials and Methods

### 2.1. Study Area

Our field experiment was conducted at Lingwu Farm (106°17′43 E, 38°07′14 N), in the upper reaches of the Yellow River basin during 2010 and 2011. In spite of the effort to promote water saving, farmers are following the old traditional way by blindly increasing the amount of chemical fertilizers, especially fertilizers N. The application rate of fertilizer N is as high as 300 kg ha^−1^, which was found after a survey of 200 farmers on over 113 ha rice field. High input of N fertilizer causes out-of-proportion soil N, P, and K. The residual N can be stored in the soil as nitrate, which flows into the Yellow River or leaches into the groundwater with the irrigation water. The soil is classified as anthropogenic-alluvial soil, which is the main soil type of Ningxia Irrigation Region, with a soil texture of 18.25% clay, 53.76% silt, and 27.99% sand (Table 1). The soil fertility is at the mid to high yield levels with a high fertilizer application rate. Soil organic matter and total N concentrations in the tillage layer (0–30 cm) of soil profile are 13.97 g kg^−1^ and 0.98 g kg^−1^ irrigation drawn from the Yellow River. The soil bulk density of the tillage layer is 1.39 g cm^−3^. Three main crop rotations occur in Ningxia Irrigation Region, namely, rice-wheat, summer maize-wheat, and rice-rice. Of these, large amount of N fertilizer has been applied into rice-rice system leach into the water bodies [16] and is therefore the focus of this study.

**Figure 1:**
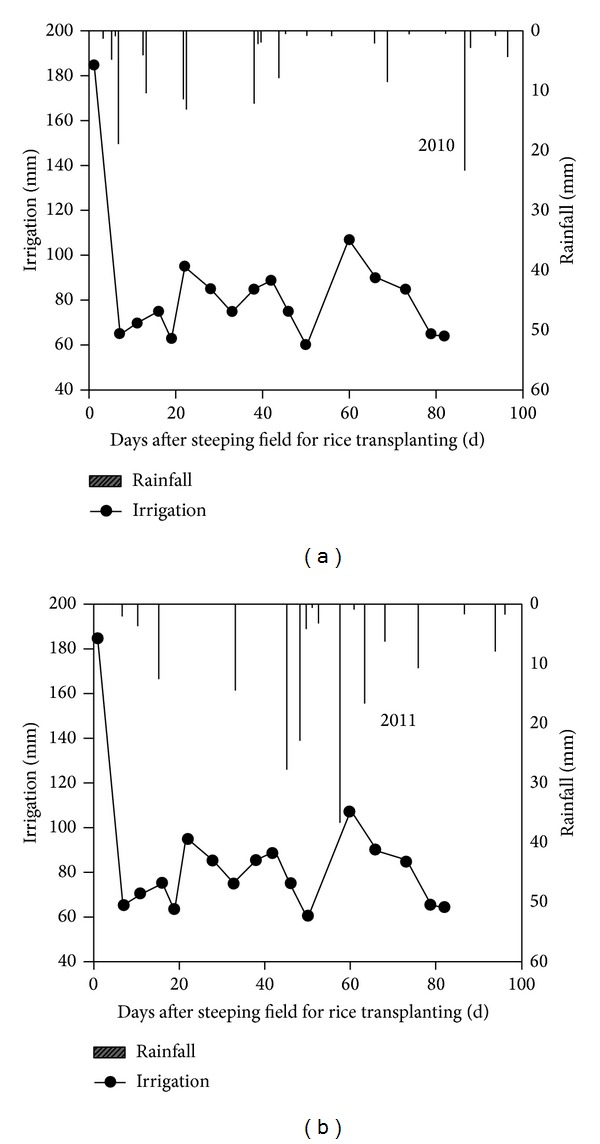
Precipitation and irrigation amount during whole growth period of rice.

Beginning on June 11, the rice field remained submerged with flooding irrigation and irrigation was on August 28, putting in 1365 m^3^ water per ha in 2010 and the precipitation is 130.9 mm (Figure 1). In 2011, beginning on May 20 and stopping irrigation on August 12 and thereafter the same as that of 2010, water per ha were put in the rice field and the precipitation is 171.1 mm during the rice-growing stage. Irrigation was mainly done during the early reproductive season from returning green to tillering and jointing-booting stages. About 80% of total irrigation water was applied before the rice heading stage. Less than 20% was used at flowering, booting, and mature stages.

### 2.2. The Field Experiment

The experiments were arranged in a randomized complete block design. The five fertilizer N treatments included (1) CK (No N fertilizer application treatment: 0 kg N ha^−1^), (2) N300 (300 kg N*·*ha^−1^, 50% used as base fertilizer, 25% as tillering fertilizer, and 25% as booting fertilizer), (3) N240 (240 kg N*·*ha^−1^, 50% used as base fertilizer, 25% as tillering fertilizer, and 25% as booting fertilizer), (4) RPN I (240 kg N*·*ha^−1^, divide the fertilizer into 3 equal amounts, each about 80 kg, used as base fertilizer, tillering fertilizer, and booting fertilizer), and (5) RPN II (240 kg N*·*ha^−1^, divide the fertilizer into 4 equal amounts, each about 60 kg, used as base fertilizer, tillering fertilizer, booting fertilizer, and panicle fertilizer). The nitrogen fertilizer is urea which contains 46% N. In all of the treatments, phosphorus (90 kg P_2_O_5_ ha^−1^) and potassium (90 kg K_2_O ha^−1^) fertilizers were ploughed into the soil tillage layer in one time before flooding the field as a basal fertilizer. The experiment design is shown in Table 2.

The rice cultivar for the 2-year experiment was 96D10. In 2010, rice seedlings were transplanted on June 14th, and the rice harvesting was on October 10th. In 2011, rice seedlings were transplanted on May 21th, and the rice harvesting was on September 21th. Each treatment had three replicates. Each plot area was 60 m^2^ and there were 15 plots in total. Trenches of 130 cm in depth were dug between the experimental plots. The field was mulched with plastic film, and double layers were installed to the inner side to prevent water interchange. Ditches and ridges were also dug. The whole experimental was separated by 2 m wide protection rows. Each treatment plot was irrigated separately, but equal amount of water was applied for all plots.

### 2.3. Sample Collection and Laboratory Analysis

During 2010 and 2011 seasons, soil water samples used for the leaching calculations were collected from lysimeters [24, 25]. Four PPR (polypropylene random) equilibrium-tension lysimeters (ETLs) (0.19 m^2^) were installed at 1.2 m below the soil surface in each treatment. The regulated vacuum system was adjusted manually several times a week to provide suction that was slightly more negative than the matric potential recorded in the surrounding bulk soil with the heat dissipation sensors. The purpose was to avoid ponding above and bypass flow around the porous plate of the lysimeters to recreate as natural a drainage pattern as possible [25].

Leaching samples were collected 1, 3, 5, 7, and 9 days after transplanting and topdressing. The subsequent sampling was conducted at 7-day intervals. We collected water sample for 21 times in 2010 and 22 times in 2011. The water samples were transferred to a plastic tube and stored at 4°C until analysis in the laboratory. At maturity, plants were removed from the field to calculate the N use efficiency (NUE) and partial factor productivity (PFP).

The NUE is defined as the ratio of the crop N uptake to the total input of N fertilizer [26]. The PFP is the ratio of grain yield to the applied N rate [27]. They were calculated as below, respectively:

(1) NUE=Nuptake  with  N  application−Nuptake  without  N  applicationNapplied ×100%,

(2) $$\begin{array}{c} \text{PFP}=\frac{Y}{{\text{N}}_{\text{applied}}}, \end{array}$$

where *Y* is grain yield, kg*·*ha^−1^, and N_applied_ is the amount of N applied, kg ha^−1^.

The N leaching loss during the rice grow stage was calculated using the following formula [25]:

(3) $$\begin{array}{c} Q_{n}=C_{n}Q_{w}. \end{array}$$

Here, *C*_*n*_ is the N concentration (TN, NH_4_^+^, and NO_3_^−^) from leaching water collected from lysimeter in the different rice growing stage, kg N m^−3^; *Q*_*w*_ is the quantity of leaching water in the corresponding stage collected from lysimeter, m^3^ *·*ha^−1^; and *Q*_*n*_ is the quantity of N leaching loss, kg ha^−1^.

Total N content of soil and rice plants was calculated by using the Kjeldahl N method, total P content using Mo-Sb colorimetric method, and total K content using flame photometry. The total N concentration of soil water sample was determined by Persulfate-UV spectrophotometry [28], and the concentrations of NH_4_^+^ and NO_3_^−^ were measured by flow injection analysis (FIA) made in France. One-way analysis of variance (ANOVA) at *α* = 0.05 probability was conducted to test the significance in different treatments. All statistiN300l analyses were performed using statistiN300l analysis System (SAS) General Linear Model procedures [29].

## 3. Results

### 3.1. Effects of Reducing and Postponing N Application on Rice Yield and Its Components

All N treatments could significantly improve rice yield compared with CK (Table 2). In 2010, the highest yield was from RPN II, which was 6737 kg/hm^2^ higher than that of CK, and then RPN I. In 2011, the highest yield was from the field of RPN I treatment, which were 5412 kg/hm^2^ higher than CK. There was no significant different between all of the N treatments in both 2010 and 2011. This indicated the nitrogen fertilizer application level of N240 kg N*·*ha^−1^ can allow current N application rates to reduce by 20% while still maintain crop yields. Because of the continuous rainfall of more than 10 days in middle and late September 2011 in Ningxia Irrigation Area, the weather was cooler than usual and rice yield reduced by about 20% in the whole irrigation area compared to the normal years, and thus the rice yield of all treatments was lower than in 2010.

The rice yield is composed of spikes number per unit area, grain number per spike, and thousand kernel weights. In 2010 and 2011, the panicles of all N treatments were higher than that of CK, but there was no significant difference among other N treatments except N300 in 2010. The grain number per panicle showed an increasing trend with N levels as follows: N300 > RPN I > N240 > RPN II > CK. The grain numbers per panicle of N300 treatment and RPN I treatment were significantly higher than those of the CK and RPN II treatments. With regard to differences among treatments, CK and RPN had the higher thousand grain weight while N300 and N240 had the lower.

### 3.2. Effects of Reducing and Postponing N Application on N Leaching Losses

High N fertilizer and irrigation amounts applied to rice on anthropogenic-alluvial soil often result in severe nitrogen loss from leaching. There is a significant correlation between N leaching losses and the N application amount. During the whole rice growing period, in each treatment, the TN leaching loss increased as the N fertilizer application amount goes up (Table 4). The total N leaching loss was 13.60 kg/hm^2^ and 9.19 kg/hm^2^ under CK treatment in 2010 and 2011, while it was 44.51 and 39.8 kg*·*ha^−1^ under the N300 treatment, respectively. The results from the two-year experiment showed that TN leaching loss under the N300 treatment was the highest and then the N240 treatment. The total N leaching loss under the RPN I treatment was substantially lower than that under N300 and N240, but there was no significant difference with RPN II. In 2010, total N leaching loss of RPN I and RPN II treatment were 30.78 and 31.32 kg*·*ha^−1^, 30.85% and 29.63% lower than the N300 treatment. In 2011, total N leaching loss of RPN I and RPN II treatment were 25.53 and 27.71 kg*·*ha^−1^, 36.31% and 30.38% lower than N300 treatment. These results indicated that RPN treatments can significantly reduce TN leaching loss. Net total N leaching loss of N fertilizers under N300, N240, RPN I, and RPN II was 30.91, 20.59, 16.18, and 17.72 kg/hm^2^ in 2010 and 30.61, 21.39, 16.16, and 18.52 kg/hm^2^ in 2011. The two-year average leaching loss of total N accounted for 10.3%, 8.75%, 6.74%, and 7.48% of applied N fertilizer.

The main leaching period of total N was delayed under the RPN treatments. Total N leaching loss under CK, N300, and N240 treatment mainly happened in the tillering stage while that of RPN I and RPN II treatments mainly happened from tillering stage to booting stage. The tillering stage was the main stage for total N leaching loss that might be due to two reasons, firstly, the higher irrigation volume at this stage for promoting tillering [30]. In addition, during this period, the basal and top dressing N inputs accounted for 50%–75% of the entire growth period with flood irrigation, which resulted in a huge leaching loss of total N.

Nitrogen loss by leaching is the major problem in rice field. Under normal conditions, soil N is leached mainly as nitrate-N (NO_3_^−^-N) which forms a major contaminant of ground water. The NO_3_^−^-N leaching losses under N300 treatment during rice growing stage were higher those that of other treatments during 2010 and 2011. The NO_3_^−^-N leaching loss of different treatments shared the same changing trend with the total N, N300 > N240 > RPN II > RPN I > CK (Table 5). The majority of leaching losses of NO_3_^−^-N under N300 and N240 treatment occurred during seedling stage and tillering stage and then booting stage while those of the RPN treatments occurred during tillering stage and booting stage and then seedling stage. The NO_3_^−^-N leaching losses of all nitrogen treatments reached the peak at tillering stage, accounting for 33.6–43.4% and 35.1–47.3% of the total loss during the rice growing stages in 2010 and 2011, respectively. The NO_3_^−^-N leaching losses of CK also mainly occurred in tillering stage, accounting for 25.4% in 2010 and 24.3% in 2011 of the total NO_3_^−^-N loss. In 2010, 35.49 and 22.08 kg NO_3_^−^-N ha^−1^ (representing about 79.7% and 71.7% of the TN leaching losses) leached below the root zone in N300 and RPN I, respectively. Thus, RPN I reduced the NO_3_^−^-N leaching loss by about 37.8% compared to N300. In 2011, 30.02 and 19.63 kg NO_3_^−^-N ha^−1^(representing about 75.4% and 70.84% of the TN leaching losses) leached out of the root zone in N300 and RPN I, respectively. In this year, RPN I reduced NO_3_^−^-N leaching losses by about 34.6% compared to N300.

Nitrogen can also be lost as NH_4_^+^-N, but the quantity is very small compared to NO_3_^−^-N. The NH_4_^+^-N losses ranked from greatest to smallest were N300 > N240 > RPN II > RPN I > CK. The major stages of NH_4_^+^-N leaching losses (Table 6) were similar to the stage of NO_3_^−^-N. Under N300 treatment, 3.58 and 3.21 kg NH_4_^+^-N ha^−1^ (representing about 8.04% and 8.07% of the TN leaching losses) leached below the root zone in 2010 and 2011, respectively. In RPN I, 2.76 and 2.94 kg NH_4_^+^-N ha^−1^ (representing about 12.5% and 14.9% of the TN leaching losses) leached out of the root zone in 2010 and 2011, respectively. From the two-year average results, RPN I reduced the NH_4_^+^-N leaching loss by about 16.1% compared to N300. There were no significant differences of the NH_4_^+^-N leaching losses at the N-fertilization level of 240 kg/hm^2^. Our results indicated that the NH_4_^+^-N leaching losses contributed very little to the total N leaching losses during rice growth stage.

### 3.3. Effects of Reducing and Postponing N Application on N Use Efficiency

Under the conditions applied in this experiment, when more N fertilizer is applied the upper soil absorbs more nitrogen but N fertilizer uses efficiency drops with increasing applied quantity of N fertilizer, and the result from two-year experiment is consistent (Table 7). Compared with CK treatment, N fertilizer application can help the rice grains and stems to absorb more nitrogen though the N fertilizer use efficiency declines with rising quantity of N fertilizer applied. The two-year average use efficiency of N fertilizer treated with N300 is merely 32.0%, and that of N fertilizer treated with N240, RPN I, and RPN II is 36.8%, 40.0%, and 39.6%, respectively, among which that of N fertilizer treated with RPN I and RPN II is higher than that with N300 by 8.0 and 7.6 percentages, and RPN I results in the highest use efficiency, 8% higher than what N300 treatment results in.

As the N applied rate increased, there was a decrease in PFP. The absolute values for PFP were higher in 2010 compared to in 2011 at all the levels of N application. The RPN I treatment had highest PFP while the N300 had the lowest both in 2010 and 2011. There was no significant difference of PFP among N240, RPN I, and RPN II. The PFP of RPN I was 20.94% and 25.38% higher than that of N300 in 2010 and 2011, respectively.

## 4. Discussions

Nitrogen loss by leaching is the major problem in rice field. Under normal conditions, soil N is leached mainly as NO_3_^−^-N which forms a major contaminant of ground water. Nitrogen can also be lost as NH_4_^+^-N, but the quantity is very small compared to the former one. A study by Yun et al. [31] revealed that N loss by leaching was not so pronounced in arid and semiarid soil, but it could be 20 to 30 kg ha^−1^ in the wet temperate zone or as high as 50 kg ha^−1^ in Europe and middle states of USA. Nitrogen enters a unique environment in anthropogenic-alluvial soil, in which losses of fertilizer N and mechanisms of losses vary greatly from those in upland situations. The study showed that the N lost was 152 to 155 kg ha^−1^, of which N lost by leaching was 78 kg ha^−1^ under the conventional fertilizer rate of 300 kg ha^−1^ [32]. It is higher than our 2-year average results of 42.16 kg ha^−1^. With the excessive application amount of fertilizer N during the flooding irrigation, NO_3_^−^-N leaching was most prevalent in the rice filed of the study area. Our results showed that the 2-year average NO_3_^−^-N leaching loss under the fertilizer rate of 300 kg ha^−1^ accounted for 77.54% of the total N loss. As SAS Institute [33] stated, a close relationship existed between the amount of NO_3_^−^-N leached and the amount of fertilizer N used in most cases. The treatment of N240, which is still higher than that for the currently grown rice cultivars in the United States, ranging from 134 to 202 kg ha^−1^ [29–31, 34–38], would maintain crop yields while substantially reducing N losses to the environment [39–42].

In Ningxia Irrigation Region, there appeared a large quantity of nitrogen leaching loss at the early stage of rice growth due to excessive N fertilizer application, improper timing of topdressing and, heavier irrigation early on. Irrigation was mainly done during the early reproductive season, and the base fertilizer and the tillering fertilizer that were applied just after about 10 days of rice seedling accounted for 80% of total N fertilizer throughout the rice growth stage; large amount of N fertilizer with flood irrigation led to massive nitrogen leaching loss [43, 44]. In the RPN I and RPN II treatments, the N fertilizer was divided into three and four equal parts and applied in three or four times; however, the resulting rice yield was similar to those with the traditional N fertilizer application and N300 treatment used by farmers, while less quantity of N fertilizer was applied at the early stage of rice growth, obviating the peak period of leaching. This led not only to increased N fertilizer use efficiency but also to reduced nitrogen leaching loss. Liu et al. [22] discovered from his study that with RPN technology, even under the condition that the quantity of N fertilizer used was 30% less compared to traditional farming, the corn yield, the surface dry matter accumulation, and N accumulation rate did not drop, and N fertilizer use efficiency rose significantly instead [20]. In this way the supply of inorganic nitrogen kept pace with crop absorption and accumulation of inorganic nitrogen in soil in the depth up to 100 cm reduced to a considerable degree, thus the field apparent loss of nitrogen mitigated. Li et al. [20] found in her investigation that, even under the condition that the quantity of N fertilizer was used 30% less than used in traditional farming, RPN did not lead to reduced wheat yield, but increased N fertilizer use efficiency with extremely low nitrogen apparent loss and enabled increasing nitrate-N's accumulation in soil in the depth 0~20 cm and reducing its accumulation in soil in the depth 20~80 cm [21]. Many researches indicated that excessive quantity of N fertilizer application, unsynchronization of demand and supply, and improper fertilization pattern are main cause of low N fertilizer use efficiency [30, 34, 35]. In this study, under the RPN I and RPN II treatment, part of nitrogen fertilizer was postponed, which maintained the sufficient supply of nitrogen need at the later stage of rice growth, and thus the rice yield remained basically unchanged when using 20% less nitrogen fertilizer. This indicated that the RPN worked to synchronize the nutrition supply and crop absorption and improved both the yield and N use efficiency. The result was consistent with Yi's research [36].

**Figure 2:**
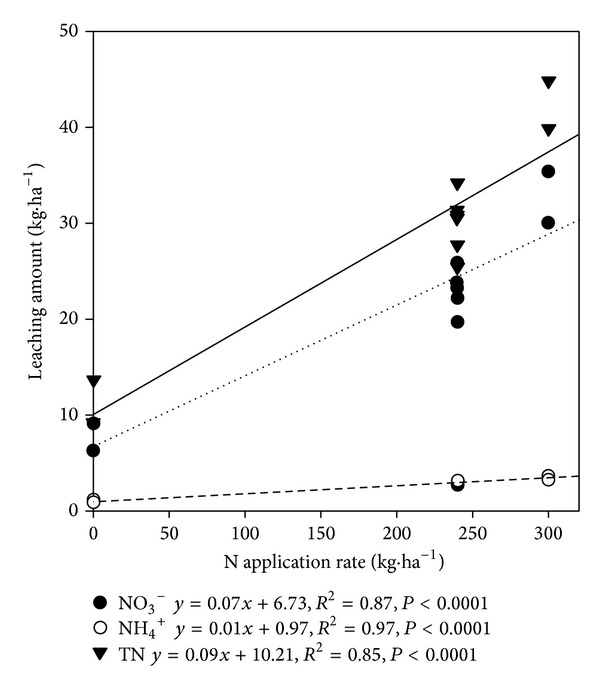
Linear regression relationships between N application rate and leaching amount of TN, NO_3_^−^ and NH_4_^+^.

Excess application of N on the field is one of the major factors of N leaching. The amount of N leached and the amount of fertilizer N used showed a close relationship [33]. The N leaching amount is in linear relationship with the fertilizer application rate (*P* < 0.05) (Figure 2). The soil texture and irrigation practice are considered other major factors of N leaching, especially in flooded irrigation area. With the excessive amount of fertilizer N application during the flooding irrigation, large amounts of N fertilizer into the land will inevitably leach N into the water bodies, and thus becoming one of the nonpoint source pollution to the Yellow River. There were about 28,500 tons of NO_3_^−^-N, 5,500 tons of NH_4_^+^-N, and 41,100 tons of TN that were lost every year due to leaching in Ningxia irrigation region. In the conditions of this experiment, total nitrogen leaching loss was 6.7%~10.3% of total N fertilizer applied and treated with various methods and the leaching is mainly in the form of NO_3_^−^-N which accounts for more than 80% in TN loss, which is consistent with the results from previous researches [14, 37]. For comparison, on the south of the Yangtze River, the predominant soil types in rice field are the yellow mud soil, red loam soil, and red purple soil. When N fertilizer was applied at a rate of 300 kg ha^−1^, no leaching of NO_3_^−^-N was found in the paddy field during rice growing season [38]. However in the Ningxia irrigation zone, a total of 18–23 times and about 1400–1600 m^3^ water per ha was put into the rice filed during the rice growing season, and the soil in the study site has a high silt percentage of 53.76%. NO_3_^−^-N leaching was most prevalent on sandy soils that received heavy irrigation. It has been shown that NO_3_^−^-N may travel with gravitational water that moves quickly down these holes during heavy flooding [33]. Zhang et al. [45] reported in their literature review that nitrate leaching was most prevalent on sandy soils that received heavy irrigation. Zhao et al. [46] reported in their literature review that irrigation practices would possibly overwhelm any recommended fertilizer application rates.

High N fertilizer and irrigation amounts applied to rice on sandy soils often result in N leaching and low use efficiency of applied fertilizer N. The RPN I treatment could allow current N use efficiency to increase. Fertilizer N use efficiency of irrigated rice is relatively low due to rapid losses of applied N through volatilization and denitrification in the soil-flood-water system [47–49]. In this 2-year study, the average N use efficiency under N300 treatment was 31.95%. The average N utilization rate reached 40.0% for RPN I treatment, which greatly reduced the amount of N fertilizer and increased the N use efficiency. It not only saved fertilizer resources, but also effectively reduced other forms of N losses. Compared with N300 treatment, RPN I could reduce 20% N application and 34.61% TN leaching loss while the yield did not significantly reduce. de Datta and Buresh [50] reported that proper timing and rate of N applications are crucial to minimize N losses [27, 28, 32, 33, 39, 40]. RPN could reduce N leaching loss because less N fertilizer was used in the early stage of rice growth and thus reduces the level of N fertilizer in the leaching solution. Because the base fertilizer, if applied excessively at one time, would accumulate nitrogen in soil and because rice needs less nitrogen at the early stage, there is great possibility for nitrogen leaching with heavy irrigation to begin [51–53]. In the later stage of rice growth when N fertilizer was applied, its root system has developed its ability to absorb more nitrogen that will cause the nitrogen concentration in the leaching solution to decline. It improved the congruence between crop N demand and the available N supply from soil and applied fertilizer, and thus increased the N use efficiency [54, 55]. The result is accordance with Cassman's report [56]. It was generally believed that under planting conditions of lower N levels (e.g., 150 kg*·*ha^−1^), the N use efficiency could be significantly improved [57]. Shi et al. [58] found that after rice flowering, while N application was higher than 200 kg*·*ha^−1^, the N translocation rate and fertilizer use efficiency would reduce with the increase of N application. High input but low utilization efficiency of chemical fertilizer N also resulted in a decrease in partial factor productivity [59, 60]. The low efficiency of fertilizer use suggests that N was not the only major limiting resource in this Region. Management interventions, particularly those that address competitive water, will likely be critical to the success of this system. A combination of careful irrigation and N management needs to be studied to improve N uptake efficiency and to minimize fertilizer N loss.

## 5. Conclusions

Achieving synchrony between N supply and crop demand without excess or deficiency is the key to optimizing tradeoffs between yield and environmental protection. The RPN I can allows current N application rates to reduce by 20% and maintain crop yields. This would significantly improve the N use efficient and thereby substantially reduce the N leaching loss.

Optimization of fertilizer application gave chance to increase nitrogen use efficiency while the rice yield was maintained. In the condition of optimized fertilizer application (nitrogen applied reduced by 20%), rice yield under RPN treatment did not decline compared with traditional fertilizer application (N300 treatment), and the two-year average yield of rice under RPN I treatment was 397 kg/hm^2^ higher than that of under N300. The average use efficiency of N300 treatment was 32.0% while the N240, RPN I, and RPN II were 36.8%, 40.0%, and 39.6%, respectively, among which RPN I treatment delivered the maximal use efficiency, 8% higher than N300 treatment.

RPN made it possible to drop nitrogen leaching loss at paddies in the irrigation region significantly. The two-year average net TN leaching loss under N300, N240, RPN I, and RPN II were 10.3%, 8.75%, 6.74%, and 7.48% of total N fertilizer applied, respectively. RPN I treatment had the minimal TN leaching loss, at 16.17 kg/hm^2^, 14.64 kg/hm^2^ less than N300 treatment. The two-year average NH_4_^+^-N leaching loss due to various treatments were 7.9%, 10.15%, 11.63%, and 10.82% and this suggested that NH_4_^+^-N was not the major nitrogen leaching loss; NO_3_^−^-N leaching loss accounted for more than 80% in TN leaching loss, regardless of the treatment. RPN I can give the same yields and lead to using less nitrogen fertilizer by 20% while it did not increase labor intensity, and thus it is one way to continue to help meet food need while minimizing the risk of negative environmental impacts. Considering the high food production, the minimum environmental threat, and the low labor intensive, we should fully take into account the RPN I by reducing fertilizer N inputs and N leaching loss. Since irrigation regimes have remarkable affects fertilizers uptake, the interaction between irrigation management and N application rate on N use efficiency in alkaline anthropogenic-alluvial soil is needed to be further studeied.

## Figures and Tables

**Table 1:** Properties of the anthropogenic-alluvial soil in the study site.

Soil depths (cm)	Bulk density (g*·*kg^−1^)	Organic matter (g*·*kg^−1^)	Total N (g*·*kg^−1^)	Porosity (%)	Soil particle size (%)		
					Clay	Silt	Sand
0–15	1.35	15.21	0.98	44.25	18.25	53.76	27.99
15–30	1.43	13.07	0.99	42.04	17.33	51.07	26.59
30–45	1.55	10.12	0.86	40.71	17.70	52.15	25.74
45–60	1.59	8.30	0.73	42.07	28.04	67.71	4.26
60–90	1.50	5.56	0.34	44.82	12.11	31.96	55.93
90–100	1.52	4.48	0.31	43.72	15.96	42.05	42.00
100–120	1.48	3.55	0.25	45.28	6.41	26.93	66.67

**Table 2:** Reducing and postponing N application experiment design.

Treatments	Total N input (kg/hm^2^)	N input/(kg/hm^2^)			
		Base fertilizer	Tillering fertilizer	Booting fertilizer	Panicle fertilizer
CK	0	0	0	0	0
N300	300	150	75	75	0
N240	240	120	60	60	0
RPN I	240	80	80	80	0
RPN II	240	60	60	60	60

Note: basic fertilizer buried in soil preparation, top dressing application in surface water.

**Table 3:** Effect of RPN on rice yield and its components.

Year	Treatment	Panicle length (cm)	Panicle number	Grain number per panicle (×10^4^ *·*ha^−1^)	1000 grain weight (g)	Average yield (kg/hm^2^)	Increased yield (kg/hm^2^)	Ratio (%)
2010	CK	16.2 b	112 c	76.7 c	25.7 a	3990 b	—	—
	N300	16.5 b	169 a	109.3 a	21.9 b	9634 a	5644	141.5
	N240	19.0 a	141 b	98.6 b	22.0 b	9631 a	5641	141.4
	RPN I	19.5 a	152 ab	105.7 a	22.8 ab	10326 a	6336	158.8
	RPN II	19.0 a	148 b	92.5 b	23.4 a	10727 a	6737	168.8

2011	CK	12.1 b	105 b	71.8 c	26.1 a	3366 b	—	—
	N300	15.4 a	147 a	111.8 a	23.7 b	8676 a	5310	157.8
	N240	15.0 a	138 a	106.4 a	23.9 b	8498 a	5132	152.5
	RPN I	15.1 a	145 a	109.7 a	24.8 a	8778 a	5412	160.8
	RPN II	14.8 a	138 a	99.4 b	24.1 ab	8612 a	5246	155.9

Figures followed by the same letters within a column for different treatments are not significantly different at the significance level *P* < 0.05 based on one-way analysis of variance (ANOVA).

**Table 4:** Total N accumulative leaching at rice growth stages (kg*·*ha^−1^).

Year	Treatment	Growth stage						
		Seedling stage	Tillering stage	Booting stage	Flowering stage	Heading stage	Harvest stage	Sum
2010	CK	1.58 d	5.11 d	2.76 b	2.42 c	1.22 c	0.50 c	13.60 d
	N300	11.24 a	19.31 a	8.45 a	3.15 b	1.47 c	0.89 bc	44.51 a
	N240	8.24 b	13.98 b	7.42 a	2.98 bc	1.12 c	0.45 c	34.19 b
	RPN I	5.14 c	10.34 c	8.16 a	3.35 b	2.47 b	1.32 b	30.78 c
	RPN II	5.22 c	7.96 c	8.24 a	4.14 a	3.72 a	2.04 a	31.32 bc

2011	CK	1.07 d	3.45 e	1.87 d	1.64 d	0.82 e	0.34 d	9.19 d
	N300	8.96 a	17.45 a	6.86 a	3.25 b	1.97 c	1.31 b	39.8 a
	N240	6.03 b	14.47 b	5.56 b	2.47 c	1.43 d	0.62 c	30.58 b
	RPN I	3.42 c	8.91 c	4.67 c	3.87 a	3.12 b	1.36 b	25.35 c
	RPN II	3.08 c	6.74 d	6.52 a	4.07 a	4.94 a	2.36 a	27.71 bc

**Table 5:** NO_3_^−^-N accumulative leaching at rice growth stages (kg*·*ha^−1^).

Year	Treatment	Growth stage						
		Seedling stage	Tillering stage	Booting stage	Flowering stage	Heading stage	Harvest stage	Sum
2010	CK	1.52 d	5.31 d	1.34 c	0.56 d	0.22 d	0.09 e	9.04 d
	N300	9.03 a	15.74 a	6.37 a	2.82 b	1.07 c	0.53 c	35.49 a
	N240	6.02 b	10.97 b	5.48 b	2.21 c	0.84 c	0.29 d	25.81 b
	RPN I	3.92 c	8.12 c	5.21 b	1.94 c	1.87 b	1.02 b	22.08 c
	RPN II	3.62 c	6.12 d	6.35 a	3.41 a	2.54 a	1.21 a	23.25 bc

2011	CK	0.81 d	2.12 e	1.45 d	1.28 e	0.52 d	0.17 d	6.35 d
	N300	7.02 a	12.84 a	5.21 a	2.52 c	1.45 c	0.98 b	30.02 a
	N240	4.83 b	10.84 b	4.76 ab	1.83 d	1.12 c	0.35 c	23.73 b
	RPN I	2.73 c	7.08 c	3.61 c	3.23 b	2.14 b	0.84 b	19.63 c
	RPN II	2.47 c	5.21 d	4.38 b	4.98 a	3.67 a	1.69 a	22.4 b

**Table 6:** NH_4_^+^-N accumulative leaching at rice growth stages (kg*·*ha^−1^).

Year	Treatment	Growth stage						
		Seedling stage	Tillering stage	Jointing stage	Flowering stage	Heading stage	Harvest stage	Sum
2010	CK	0.15 a	0.35 c	0.15 d	0.11 c	0.26 b	0.06 d	1.09 c
	N300	0.18 a	1.09 a	0.77 a	0.53 a	0.67 a	0.34 b	3.58 a
	N240	0.14	1.23 a	0.71 ab	0.27 b	0.23 b	0.28 c	2.86 b
	RPN I	0.14 a	0.82 b	0.62 b	0.25 b	0.62 a	0.31 bc	2.76 b
	RPN II	0.13 a	0.84 b	0.41 c	0.43 a	0.57 a	0.43 a	2.81 b

2011	CK	0.08 c	0.23 d	0.17 c	0.12 d	0.16 d	0.08 d	0.84 b
	N300	0.18 a	1.27 a	0.65 ab	0.44 b	0.48 c	0.19 b	3.21 a
	N240	0.17 a	1.12 a	0.68 ab	0.32 c	0.72 b	0.14 c	3.15 a
	RPN I	0.15 ab	0.89 b	0.71 a	0.34 bc	0.64 b	0.21 b	2.94 a
	RPN II	0.10 c	0.64 c	0.51 b	0.62 a	0.81 a	0.36 a	3.04 a

**Table 7:** Effects of RPN on NUE and PFP.

Year	Treatment	Straw N uptake (kg/hm^2^)	Grain N uptake (kg/hm^2^)	Total N uptake (kg/hm^2^)	NUE (%)	PFP (kg grain kg^−1^N)
2010	CK	37.0	43.5	80.5 b	—	—
	N300	62.2	113.3	175.5 a	31.7 b	32.11 b
	N240	61.8	108.3	170.1 a	37.3 a	40.13 a
	RPN I	63.2	114.6	177.8 a	40.5 a	43.03 a
	RPN II	68.7	110.4	179.1 a	41.1 a	44.7 a

2011	CK	16.2	37.4	53.6 b	—	—
	N300	54.3	95.8	150.1 a	32.2 b	28.92 b
	N240	50.4	90.2	140.6 a	36.3 a	35.41 a
	RPN I	53.6	94.8	148.4 a	39.5 a	36.58 a
	RPN II	49.5	95.3	144.8 a	38.0 a	35.88 a

## References

[1] Bao SD (2000). *Soil and Agricultural Chemistry Analysis*.

[2] Bergstrom L (1990). Use of lysimeters to estimate leaching of pesticides in agricultural soils. *Environmental Pollution*.

[3] Brye KR, Norman JM, Bundy LG, Gower ST (1999). An equilibrium tension lysimeter for measuring drainage through soil. *Soil Science Society of America Journal*.

[4] Burgos NR, Norman RJ, Gealy DR, Black H (2006). Competitive N uptake between rice and weedy rice. *Field Crops Research*.

[5] Canfield DE, Glazer AN, Falkowski PG (2010). The evolution and future of earth’s nitrogen cycle. *Science*.

[6] Cao XM, Liu H (2004). Quality analysis of groundwater in Ningxia. *Ningxia Engineering and Technology*.

[7] Cassman KG, Kropff MJ, Yan ZD (1994). A conceptual framework for nitrogen management of irrigated rice in high-yield environments. *Hybrid Rice Technology: New Developments and Future Prospects*.

[8] Cassman KG, de Datta SK, Amarante ST, Liboon SP, Samson MI, Dizon MA (1996). Long-term comparison of the agronomic efficiency and residual benefits of organic and inorganic nitrogen sources for tropical lowland rice. *Experimental Agriculture*.

[9] Conrad Y, Fohrer N (2009). Modelling of nitrogen leaching under a complex winter wheat and red clover crop rotation in a drained agricultural field. *Physics and Chemistry of the Earth*.

[10] Cui Z, Zhang F, Chen X (2008). On-farm estimation of indigenous nitrogen supply for site-specific nitrogen management in the North China plain. *Nutrient Cycling in Agroecosystems*.

[11] de Datta SK, Buresh RJ (1989). Integrated nitrogen management in irrigated rice. *Advances in Soil Science*.

[12] Fixen PE, West FB (2002). Nitrogen fertilizers: meeting contemporary challenges. *Ambio*.

[13] Zhang F, Cui Z, Wang J, Li X C (2007). Current status of soil and plant nutrient management in China and improvement strategies. *Chinese Bulletin of Botany*.

[14] Heffer P (2009). *Assessment of Fertilizer Use by Crop at the Global Level: 2006/07–2007/08*.

[15] Ju XT, Xing GX, Chen XP (2009). Reducing environmental risk by improving N management in intensive Chinese agricultural systems. *Proceedings of the National Academy of Sciences of the United States of America*.

[16] Keeney DR (1982). Nitrogen management for maximum efficiency and minimum pollution. *Nitrogen in Agricultural Soils*.

[17] Kenna MR, Horst GL (1993). Turfgrass water conservation and quality. *International Turfgrass Society Research Journal*.

[18] Lea PJ, Azevedo RA (2006). Nitrogen use efficiency. 1: uptake of nitrogen from the soil. *Annals of Applied Biology*.

[19] Li QK, Li HE, Hu YW, Sun J (2008). Nitrogen loss in Qingtongxia irrigation area. *Journal of Agro-Environment Science*.

[20] Li WJ, Xia YQ, Yang XY, Guo M, Yan XY (2011). Effects of applying nitrogen fertilizer and fertilizer additive on rice yield and rice plant nitrogen uptake, translocation, and utilization. *Chinese Journal of Applied Ecology*.

[21] Liang XQ, Chen YX, Li H, Tian G, Yu QG (2006). Effect of rainfall intensity and rain-fertilization interval on N export by runoff in oilseed rape land. *Journal of Soil and Water Conservation*.

[22] Liu J, You L, Amini M (2010). A high-resolution assessment on global nitrogen flows in cropland. *Proceedings of the National Academy of Sciences of the United States of America*.

[23] Liu T, Tang J, Jiang S (2012). Effect of postponing nitrogen fertilizer application on yield and nitrogen using efficiency of super rice Yangliangyou6. *Journal of Northeast Agricultural University*.

[24] (2012). *Ningxia Statistical Yearbook*.

[25] Ningxia Water Conservancy Bureau (2008). *Ningxia Water Resource Bulletin No. 22*.

[26] Pei XX, Wang XB, He P (2009). Effect of postponing N application on soil N supply, plant N uptake and utilization in winter wheat. *Plant Nutrition and Fertilizer Science*.

[27] Peng S, Garcia FV, Laza RC, Sanico AL, Visperas RM, Cassman KG (1996). Increased N-use efficiency using a chlorophyll meter on high-yielding irrigated rice. *Field Crops Research*.

[28] Prunty L, Greenland R (1997). Nitrate leaching using two potato-corn N-fertilizer plans on sandy soil. *Agriculture, Ecosystems and Environment*.

[29] Yu T, Chen JS (2004). Impacts of the agricultural development on the water quality and nitrogen pollution of the Yellow River-case of Ningxia irrigation area. *Journal of Arid Land Resources & Environment*.

[30] Yi J (2011). *Study on the characteristics of nitrogen migration in rice fields in Ningxia irrigation region [M.S. thesis]*.

[31] Yun F, Li Y, Yang JN (2005). Investigation on simulation of dynamic distribution of COD and ammonia-nitrogen pollution in Ningxia segment of the Yellow River. *Journal of Ningxia University: Natural Science Edition*.

[32] Riley WJ, Ortiz-Monasterio I, Matson PA (2001). Nitrogen leaching and soil nitrate, nitrite, and ammonium levels under irrigated wheat in Northern Mexico. *Nutrient Cycling in Agroecosystems*.

[33] SAS Institute (1990). *SAS User’s Guide: Statistics*.

[34] Yang SJ, Zhang AP, Yang ZL, Yang SQ (2009). Agricultural non-point source pollution in Ningxia irrigation district and preliminary study of load estimation methods. *Scientia Agricultura Sinica*.

[35] Yi Q, Zhao SC, Zhang XZ, Yang L, Xiong GY, He P (2012). Yield and nitrogen use efficiency as influenced by real time and site specific nitrogen management in two rice cultivars. *Plant Nutrition and Fertilizer Science*.

[36] Zebarth BJ, Drury CF, Tremblay N, Cambouris AN (2009). Opportunities for improved fertilizer nitrogen management in production of arable crops in eastern Canada: a review. *Canadian Journal of Soil Science*.

[37] Zhang H, Yang Z, Luo L (2011). The feature of N_2_O emission from a paddy field in irrigation area of the yellow river. *Acta Ecologica Sinica*.

[38] Zhang Q, Zhang H, Yi J (2010). The fate of fertilizer-derived nitrogen in a rice field in the Qingtongxia irrigation area. *Acta Scientiae Circumstantiae*.

[39] Shi Y, Yu ZW (2006). Effects of nitrogen fertilizer rate and ratio of base and topdressing on yield of wheat, content of soil nitrate and nitrogen balance. *Acta Ecologica Sinica*.

[40] Turner TR, Hummel NW (1992). Nutritional requirements and fertilization. *Turfgrass-Agronomy Monograph*.

[41] Vitousek PM, Naylor R, Crews T (2009). Nutrient imbalances in agricultural development. *Science*.

[42] Wang Y, Yang S (2011). The nitrate-nitrogen leachingamount in paddy winter-spring fallow period. *Acta Ecologica Sinica*.

[43] Xiaoliang X, Zuliang S, Qi J, Tingbo D, Dong J, Weixing C (2010). Effects of nitrogen fertilization on spatial-temporal distributions of soil nitrate and nitrogen utilization in wheat season of rice-wheat systems. *Acta Pedologica Sinica*.

[44] Xing CH, Zhang YS, Lin XY, Du ST, Yu CY (2006). Study on decreasing ammonia volatilization and leaching rates by NDSA fertilization method. *Journal of Zhejiang University: Agriculture and Life Sciences*.

[45] Zhang Q, Yang Z, Zhang H, Yi J (2012). Recovery efficiency and loss of 15N-labelled urea in a rice-soil system in the upper reaches of the Yellow River basin. *Agriculture, Ecosystems and Environment*.

[46] Zhao SC, Pei XX, He P (2010). Effects of reducing and postponing nitrogen application on soil N supply, plant N uptake and utilization of summer maize. *Plant Nutrition and Fertilizer Science*.

[47] Tang XL, Mu XM, Shao HB, Wang HY, Brestic M (2014). Global plant-responding mechanisms to salt stress: physiological and molecular levels and implications in biotechnology. *Critical Reviews in Biotechnology*.

[48] Xu G, Shao HB, Sun JN, Chang SX (2012). Phosphorus fractions and profile distribution in newly formed wetland soils along a salinity gradient in the Yellow River Delta in China. *Journal of Plant Nutrition and Soil Science*.

[49] Sun JN, Xu G, Shao HB, Xu SH (2012). Potential retention and release capacity of phosphorus in the newly formed wetland soils from the Yellow River Delta, China. *Clean—Soil, Air, Water*.

[50] Xu G, Lv Y, Sun J, Shao H, Wei L (2012). Recent advances in biochar applications in agricultural soils: benefits and environmental implications. *Clean—Soil, Air, Water*.

[51] Shao HB, Cui BS, Bai JH (2012). Wetland ecology in China. *Clean—Soil, Air, Water*.

[52] Yan K, Chen P, Shao H (2012). Responses of photosynthesis and photosystem II to higher temperature and salt stress in Sorghum. *Journal of Agronomy and Crop Science*.

[53] Yan K, Chen P, Shao H, Shao C, Zhao S, Brestic M (2013). Dissection of photosynthetic electron transport process in sweet Sorghum under heat stress. *PLoS ONE*.

[54] Yan K, Shao H, Shao C (2013). Physiological adaptive mechanisms of plants grown in saline soil and implications for sustainable saline agriculture in coastal zone. *Acta Physiologiae Plantarum*.

[55] Wei L, Xu G, Shao H, Sun J, Chang SX (2013). Regulating environmental factors of nutrients release from wheat straw biochar for sustainable agriculture. *Clean—Soil, Air, Water*.

[56] Yan K, Chen P, Shao HB, Zhao SJ (2013). Characterization of photosynthetic electron transport chain in bioenergy crop Jerusalem artichoke (Helianthus tuberosus L.) under heat stress for sustainable cultivation. *Industrial Crops and Products*.

[57] Huang Z, Zhao L, Chen D (2013). Salt stress encourages proline accumulation by regulating proline biosynthesis and degradation in Jerusalem Artichoke plantlets. *PLoS ONE*.

[58] Shi Y, Ge Y, Chang J, Shao H, Tang Y (2013). Garden waste biomass for renewable and sustainable energy production in China: potential, challenges and development. *Renewable and Sustainable Energy Reviews*.

[59] Jin S, Liu L, Liu Z, Long X, Shao H, Chen J (2013). Characterization of marine Pseudomonas spp. antagonist towards three tuber-rotting fungi from Jerusalem artichoke, a new industrial crop. *Industrial Crops and Products*.

[60] Shao HB, Chu LY (2013). Some progress in the study of plant-soil interactions in China. *Plant Biosystems*.

